# Ligands of HMG-like dorsal switch protein 1 of *Spodoptera exigua* leads to mortality in diamondback moth, *Plutellaxylostella*

**DOI:** 10.1016/j.heliyon.2024.e27090

**Published:** 2024-03-09

**Authors:** Md. Mahi Imam Mollah

**Affiliations:** a Department of Entomology, Patuakhali Science and Technology University, Dumki, 8602, Patuakhali, Bangladesh; b Department of Plant Medicals, College of Life Science, Andong National University, Andong, Republic of Korea

**Keywords:** *Plutella xylostella*, Dorsal switch protein 1 (DSP1), HMG boxes, Ligands, Mortality

## Abstract

HMG-like dorsal switch protein 1 (DSP1) is the insect homolog of the high mobility group box 1 (HMGB1) protein of the vertebrates. Previous studies confirmed DSP1 in *Spodoptera exigua*, *Tenebrio molitor*, and *Aedes albopictus,* and were analyzed for their immune roles, survivability, and binding affinity with entomopathogenic bacterial metabolites. The present study aimed to predict, and confirm DSP1 in diamondback moth, *Plutella xylostella* along with the effect of *Spodoptera exigua* DSP1 ligands in the survivability of this insect. DSP1 of *Plutella xylostella* (Px-DSP1) consists of 465 amino acids (AA). Phylogeny analysis showed that Px-DSP1 clustered with other Lepidopteran insects where each insect order clustered separately. Domain analysis showed that like other insects, Px-DSP1 contains two HMG boxes (Box A and Box B), one coiled-coil (CC), five Q-rich low complexity (LC), and an acidic tail (AT). Px-DSP1 was expressed in each developmental stage and tissue. The highest expression was in L4 larvae and fat body tissues. Thermal shift assay (TSA) showed the binding affinity of 3-Ethoxy-4-Methoxyphenol (EMP), Phthalimide (PM), and *o*-Cyanobenzoic acid (CBA) to rDSP1 of *Spodoptera exigua*. Mortality bioassay showed that all these metabolites were toxic against *P. xylostella* larvae. Among these, EMP was more toxic providing more than 65% mortality at 500 ppm concentration. However, PM and CBA also showed more than 60 and 50% mortality, respectively at 500 ppm concentration. We assume that like Se-DSP1, these compounds also bind with Px-DSP1 which leads to the inhibition of DSP1-mediated immunity and impose the mortality of *Plutella xylostella* larvae.

## 1 Introduction

Insects have prominent innate immunity but no acquired immune system [1]. The immunity in insects is governed by many immune proteins including peptidoglycan recognition proteins [2], Arachidonic acid, Antimicrobial peptides, Phospholipase A_2_, Prophenol oxidase, etc. [3]. Recent studies reported Dorsal switch protein 1 (DSP1) as a damage-associated molecular pattern (DAMP) with immune functions in several insects including *Spodoptera exigua* [4], *Tenebrio molitor* [5], and *Aedes albopictus* [6]. It is the insect homolog of the high mobility group box 1 (HMGB1) protein of the vertebrates where it plays the role of a DAMP [7]. Both HMGB1 and DSP1 contain HMG boxes A, HMG box B, and an acidic tail although DSP1 has additional Q-rich Low complexity (LC) and Coiled-coil (CC) domains which are variable in number and positions [4,8]. However, the specific function of these additional domains is yet to be known.

DSP1 is a nuclear protein that remains in the nucleus but is released from there upon stimulation by pathogens or stress and acts as a DAMP molecule [9]. In the infected insects, DSP1 played significant immune roles by producing antimicrobial peptides (AMPs), phenol oxidase (PO), phospholipase A_2,_ and nodules [4,9]. To introduce these immune functions, DSP1 follows the Toll-Spatzle pathway [9,10]. Toll-Spatzle is an immune pathway in the insect system that activates immune responses, especially AMP production [11], and PLA_2_ synthesis [12] against Gram-positive bacteria. The entrance of DSP1 in the Toll-Spatzle pathway is yet to be known. However, the Spatzle processing enzyme among the three proteolytic cascades of *T. molitor* is involved in activating this pathway [10]. Insects are very susceptible to entomopathogenic bacteria (EPB) that kill the insects sharply by secreting some toxic chemicals [13,14], several immune suppressors that inhibit AMP synthesis, PLA_2_ activity, nodulation, and PO activity [15,16], and apoptotic materials [17]. Metabolites of the EPB also have a binding affinity with immune proteins like DSP1 or sPLA_2_ which interrupts the immune functions of DSP1 [9,18]. Mortality bioassay with these compounds against *S. exigua* larvae showed significant mortality [9]. In this study, we hypothesized that these metabolites may also affect the survivability of the diamondback moth, *Plutella xylostella,* and impose mortality on this insect by binding to DSP1, a crucial insect immune mediator. For these, we confirmed DSP1 in *P. xylostella*, checked its identity and similarity with Se-DSP1, conducted a binding assay, and finally mortality bioassay of *P. xylostella* with the DSP1 ligand compounds. This study suggests a possible method for screening and developing potential insecticides against diamondback moths.

## 2 Materials and methods

### 2.1 Insect rearing and bacteria culture

Diamondback moth larvae were collected from the Chinese cabbage (*Brassica rapa*) fields in Andong, Korea, and reared on Chinese cabbage leaves in laboratory conditions while the moths were provided with sucrose solution (10%). Rearing conditions were: 25 ± 2 °C temperature, 60 ± 5% relative humidity, 8 h of darkness, and 16 h of daylight. In this condition, larvae underwent fourth larval molts (L1-L4).

### 2.2 Chemical and reagents

Identified bacterial metabolites such as Phthalimide (PM), *o*-Cyanobenzoic acid (CBA), and 3-Ethoxy-4-Methoxyphenol (EMP) were obtained from Sigma-Aldrich Korea (Seoul, Korea) and dimethylsulfoxide (DMSO) was used to dissolve these. Trizol reagent was collected from Invitrogen Korea (Seoul, Korea) and used for RNA extraction. Chloroform and Ethanol were collected from Sigma-Aldrich Korea (Seoul, Korea). Phosphate-buffered saline (PBS, pH 7.4) was prepared in the laboratory with 100 mM phosphoric acid and 0.7% sodium chloride. Anticoagulant buffer (ACB, pH 4.5) containing 186 mM NaCl, 17 mM Na2EDTA, and 41 mM citric acid was prepared in the laboratory.

### 2.3 Sequence analysis and bioinformatics

A DSP1 sequence of *P. xylostella* was obtained from GenBank (accession number XM_038111967). Analysis for the phylogenetic study was performed with the MEGA6 supported by the Clustal W program (www.ebi.ac.uk). To support branches, bootstrapping values were obtained with 1000 repetitions. GenBank-related information on all the genes used for phylogeny analysis is described in Supplementary information (Table S2). Search programs including SMART (http://smart.embl-heidelberg.de/) and Pfam (http://pfam.xfam.org) were used for protein domain prediction.

### 2.4 Extraction of RNA, RT-PCR and RT-qPCR

Extraction of total RNA, preparation of cDNA, and RT-qPCR were carried out following Cooper et al. [19]. Briefly, total RNA was extracted from eggs (50), L1 larvae (30), L2 larvae (20), L3 larvae (10), L4 larvae (5), pupae (5), and adults (2) with Trizol reagent (Invitrogen, CA, USA). For tissue, hemocyte was collected from 50 L4 larvae, fat body and midgut from 10 L4 larvae, and epidermis from 5 L4 larvae. Following DNase treatment, the total RNA was used as the template for cDNA synthesis with RT-Premix oligo-dT (5′‐CCAGTGAGCAGAG.

TGACGAGGACTCGAGCTCAAGCT(16)‐3′; Intron Biotech.) in a reaction volume of 20 μl. This cDNA was used as the template for RT-PCR with the gene-specific primers (**S1 Table)** where an initial heat treatment (94 °C) was applied for 2 min, then 35 cycles of each denaturing (94 °C), annealing (65 °C), and extension temperature (72 °C) was applied where the duration was 30 s for each step. RT-qPCR analysis was carried out using primers that are specific to genes (Table S1) with an RT-qPCR System (Step One, Applied Biosystem, Marsiling, Singapore). The initial heat was 95 °C for 10 min, denaturation at 95 °C for 15 s, annealing based on primer for 30 Sec, and extension temperature at 72 °C for 30 s, and each denaturation, annealing, and extension repeated by 40 cycles. Finally, 1 cycle of 95 °C (15 s), annealing (1 min), and dissociation at 95 °C for 15 s was performed. As a reference gene, the ribosomal RL32 gene was used. mRNA expression was analyzed using a comparative CT method described by Livak and Schmittgen [20]. Every experiment was replicated three times with individual samples.

### 2.5 Binding of bacterial compounds to recombinant DSP1 of S. exigua

To evaluate the binding affinity of bacterial metabolites with rSe-DSP1, a thermal shift assay was carried out using a Thermal Shift dye kit (Applied Biosystem, Foster City, CA, USA) following Simeonov [21] and Mollah et al. [9]. Briefly, the reaction mixture (20 μL) contains protein thermal shift dye (2.5 μL), protein thermal shift buffer (5 μL), rSe-DSP1 protein (10 μL, 500 ng), and test bacterial compound (2.5 μL) ensuring final density of 0, 2, 4, 6, and 8 μM. A melting curve experiment was conducted with a Step One real-time PCR system (Applied Biosystems, Foster City, CA, USA). The thermal profile was obtained by heat treatment at 25 °C for 2 min and 99 °C for 2 min. Melting temperatures resulting from the heat treatment were plotted with SigmaPlot 10.0 (Systat Software, San Jose, CA, USA). The dissociation constant (Kd) was calculated using the ligand binding equation category.

### 2.6 Insecticidal bioassays

To determine the toxicities of the bacterial metabolites, a bio-assay was conducted using *P. xylostella* larvae as a test insect. For this, oral administration was adopted where the cabbage leaf (2 cm^2^) was dipped into the metabolite solution of different concentrations (0, 0.01, 0.1, 1, 10, 100, 250, and 500 ppm). Treated leaves were then provided to 6 h unfed L2 larvae in a 9 cm diameter Petri dish. The treated larvae were kept in the insect rearing room having 25 ± 2 °C temperature and 60 ± 5% relative humidity. Following 24 h feeding, treated leaves were replaced with fresh cabbage leaves. Each test concentration was replicated thrice and each replication included 10 larvae. Mortality was recorded every 24 h. Dead larvae were confirmed by touching. Mortality data was recorded up to 96 h after treatment. The solvent used for dissolving the compound was used as a control (0.0 ppm). The compounds were dissolved in a solvent prepared by mixing 750 mL sterilized water, 24.8 mL DMSO, and 0.2 mL Triton X-100. Every treatment was replicated thrice.

### 2.7 Statistical analysis

All the continuous variable data were subjected to a one-way analysis of variance (ANOVA) using the SAS program [22]. Mortality data were transformed using an appropriate transformation system. Means were separated using the least significant difference (LSD) test at Type I error = 0.05. The median lethal dose (LD_50_) was calculated using Probit analysis of the EPA Probit Analysis Program, ver. 1.5 (Environmental Protection Agency, USA). Each treatment was replicated three times with individual samples.

## 3 Results

### 3.1 Prediction of P. xylostella DSP1 (Px-DSP1) and comparison with S. exigua DSP1

**Fig. 1:**
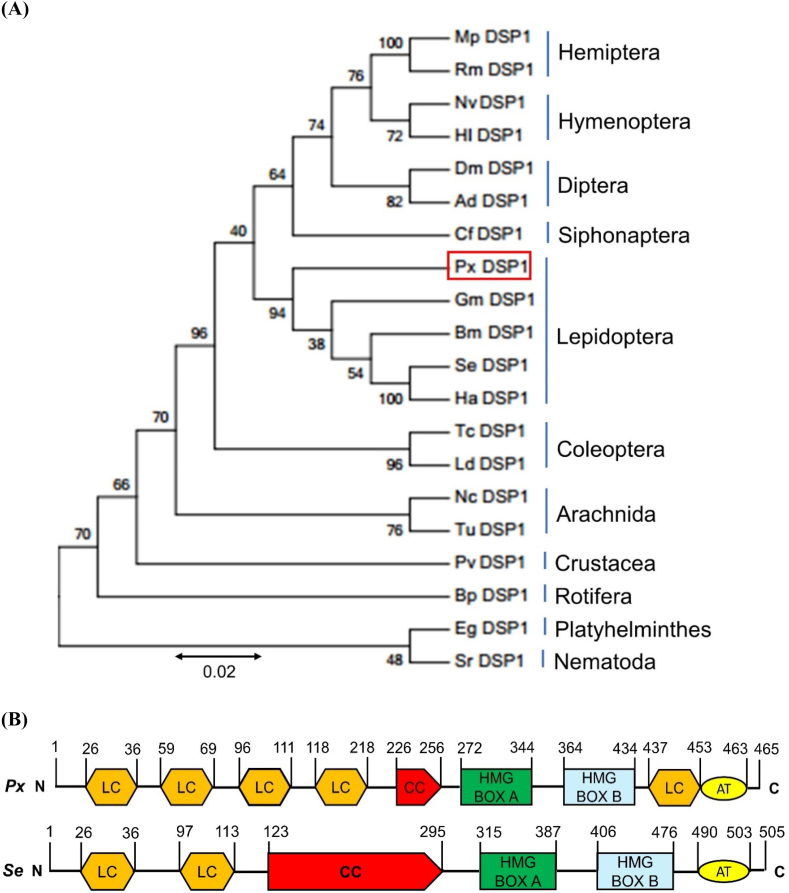
**Molecular characterization of *Plutella xylostella* DSP1 (Px‐DSP1).** (**A**) Phylogenetic analysis of Px‐DSP1 with other dorsal switch protein 1 (DSP1) genes using MEGA6 and Clustal W program. Bootstrapping values were obtained with 1000 repetitions to support branching and clustering. Amino acid sequences of DSP1 were retrieved from GenBank with accession numbers shown in Table S2. (**B**) Domain analysis of Px‐DSP1 and Se-DSP1 for comparative study. Domains were predicted using Prosite (https://prosite.expasy.org/) and SMART protein (http://smart.embl-heidelberg.de/).

DSP1 of *P. xylostella* was predicted from the transcriptome (GenBank accession number: XP_011560328.1). Its ORF consisted of 1395 bp encoding 465 amino acids. Px-DSP1 shared 73.64, 73.30 and 75.86% protein sequence similarities with the DSP1 genes of *Spodoptera exigua*, *Drosophila melanogaster,* and *Tenebrio molitor*, respectively (Fig. 1A). Phylogeny analysis revealed that Px-DSP1 was closely related to other lepidopterans within an insect cluster (Fig. 1A). Like HMGB1 of *Homo sapiens*, DSP1 of diamondback moth also have two HMG boxes (Box A and B), and an acidic tail (Fig. 1B). In addition, like other insects DSP1*,* low complexity (LC), and coiled coil (CC) domains are also present (Fig. 1B). However, the number, size, and position of LC or CC varies among the insect species.

HMG boxes of DSP1 are crucial for their functions. DSP1 of *P. xylostella* and *S. exigua* exhibited higher sequence similarity in box B providing 95.77% identity and 97.26% similarity (68 of 71 AA are similar) (Fig. 3B) compared to box A providing 93.15% identity and 97.18% similarity (68 of 73 AA are similar) (Fig. 3C).

### 3.2 Expression profile of DSP1 in P. xylostella

**Fig. 2:**
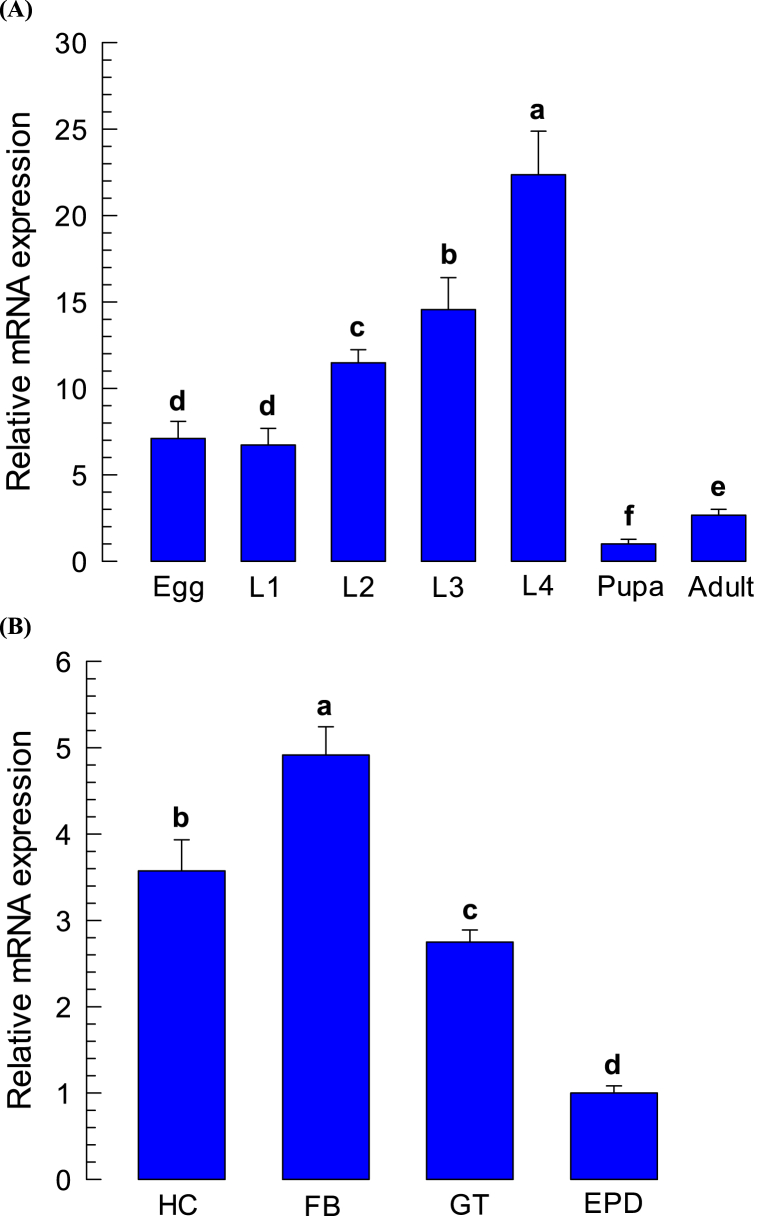
**Expression profiles of *Plutella xylostella* DSP1 (Px‐DSP1).** (**A**) Expression patterns of Px‐DSP1 at different developmental stages: egg, all larval instars (L1, L2, L3, L4), pupa, and adult. (**B**) Expression patterns in different tissues of L4 larvae: hemocyte (HC), fat body (FB), midgut (GT) and epidermis (EPD). Expression was analyzed using Applied Bioscience RT qPCR machine based on Δ-CT method. A ribosomal gene, RL32 was used as a reference gene. Each treatment was replicated three times with independent sample preparations. Different letters above the bar indicate significant differences among means at type I error = 0.05 (LSD test).

In every developmental stage, *Px-DSP1* was expressed considerably, and the expression level varies significantly (*P < 0.00014*) among the developmental stages. A high level of expression was found at the L4 larval stage followed by the L3 larvae whereas the lowest expression was in the pupal (Fig. 2A). *Px-DSP1* was expressed in all the tested tissues including hemocytes, fat body, gut, and epidermis of L4 larvae. Among the tissues, the level of *Px-DSP1* expression also varies significantly (*P < 0.00012*) where maximum expression was in fatbody and minimum in the epidermis (Fig. 2B).

**Fig. 3:**
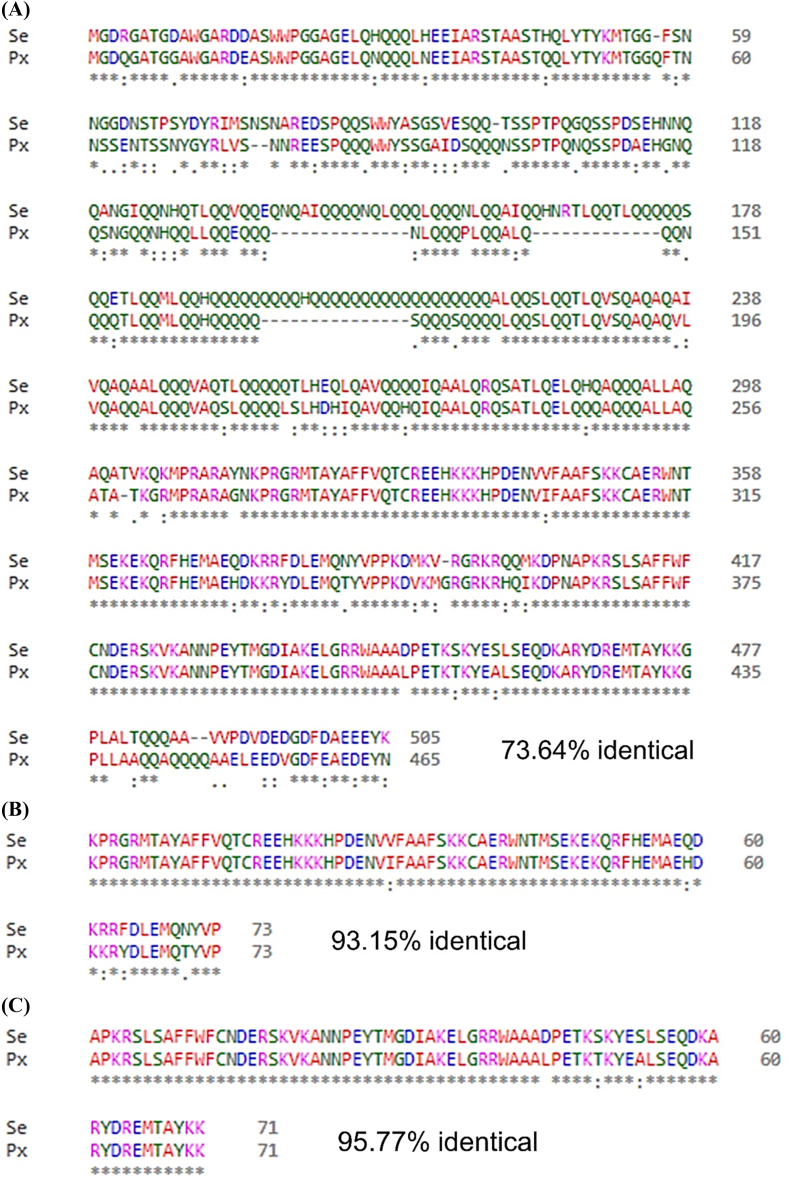
**Comparative study of *P. xylostella* and *S. exigua* DSP1 protein sequences**. (**A**) Similarity assessment of *P. xylostella* DSP1 and *S. exigua* DSP1 protein sequences. (**B**) Similarity assessment of HMG box A of *P. xylostella* and *S. exigua*. (**C**) Similarity assessment of HMG box B of *P. xylostella* and *S. exigua*. Before similarity analysis, sequences were aligned using the Clustal Omega Multiple sequence alignment program of the European Bioinformatics Institute (https://www.ebi.ac.uk). Similarity and identity of the sequences were assessed by Sequence Manipulation Suite (SMS) program (https://www.bioinformatics.org) and NCBI-protein blast (https://blast.ncbi.nlm.nih.gov).

### 3.3 Bacterial metabolites have a binding affinity with rSe-DSP1

**Fig. 4:**
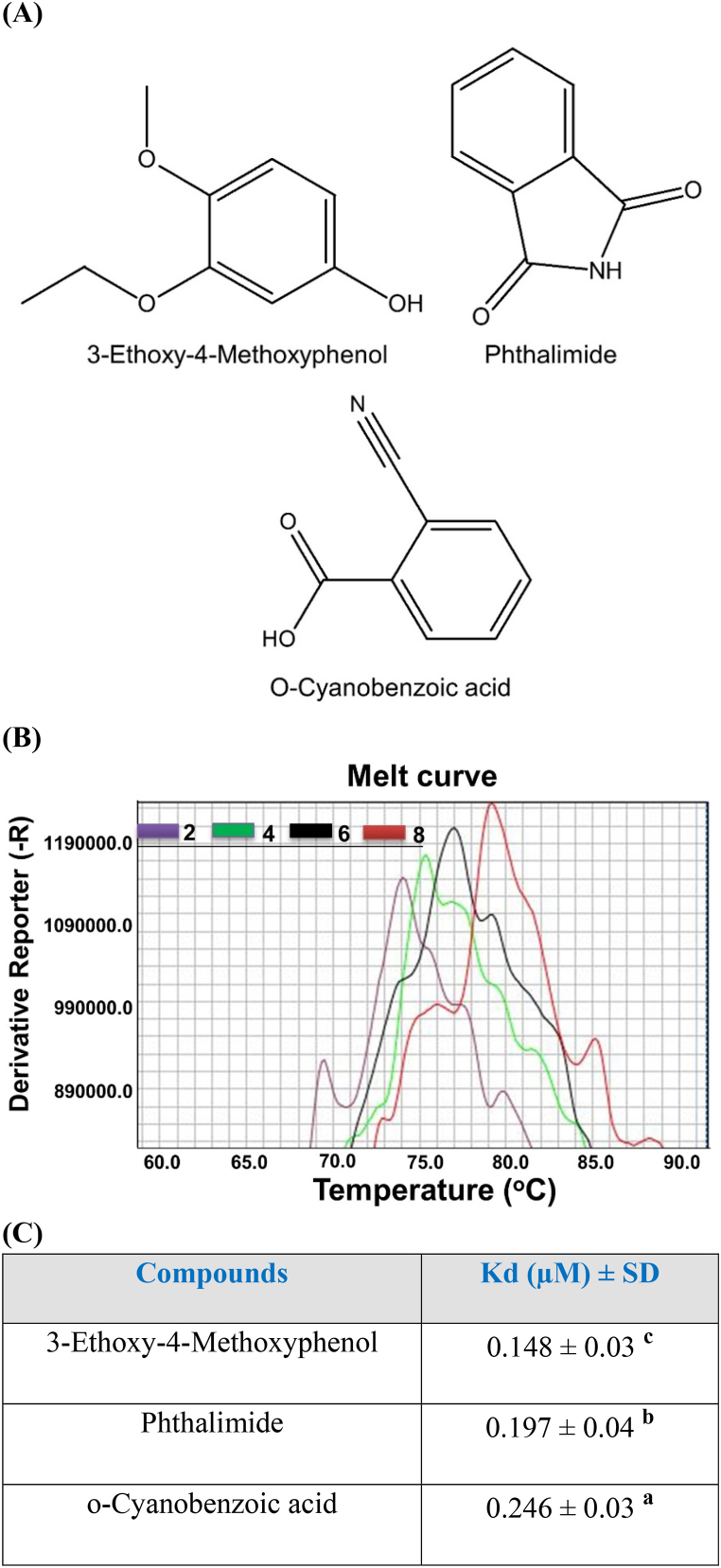
**Prediction of DSP1 binding compounds (ligands) from bacterial metabolites.** (**A**) Structure of the identified bacterial metabolites. Chemoffice 2001 was used for drawing the structures. (**B**) Binding affinity assessment by thermal shift assay (TSA) (**C**) Dissociation constant (Kd) of the DSP1 ligands. Metabolites were purified from *Xenorhabdus hominickii* culture broth after organic extraction with butanol using GC-MS analysis in another study [9]. Different concentration of the metabolites was mixed with Thermal shift buffer, dye, and recombinant protein of *S. exigua*. A melting curve experiment was conducted with this mixture using a Step One real-time PCR system. The thermal profile was obtained by heat treatment at 25 °C for 2 min and 99 °C for 2 min. The dissociation constant (Kd) was calculated using the ligand binding equation category of SigmaPlot 10.0. Each treatment was replicated three times. Different letters following standard deviation (SD) indicate significant differences among means at Type I error = 0.05 (LSD test).

A previous study identified some bacterial metabolites from the entomopathogenic bacteria *Xenorhabdus hominickii* based on their immunosuppressive activity [23]. In the present study, the phenolic compounds among the identified compounds having salicylic acid-like structures were checked for their binding affinity with a universal insect immune protein, DSP1 (Fig. 4A). Salicylic acid (SA) is a universal ligand of the human HMGB1 protein [24]. For this, a recombinant DSP1 protein (rSe-DsP1) was constructed against the lepidopteran insect *S. exigua* DSP1. When these compounds were checked for binding intensity to rSe-DSP1, a high level of binding affinities at the micromolar range was observed (Fig. 4B). However, the highest binding affinity was exhibited by 3-ethoxy-4-methoxyphenol (EMP) which was followed by Phthalimide (PM) and *o*-cyanobenzoic acid (CBA) (Fig. 4C). Although having a similar chemical structure, EMP is more efficient than SA for binding to rSe-DSP1. SA is also a ligand of rSe-DSP1 that inhibits the immune responses of *S. exigua* [9].

### 3.4 Inhibition of DSP1-induced immunity caused mortality in P. xylostella larvae

**Fig. 5:**
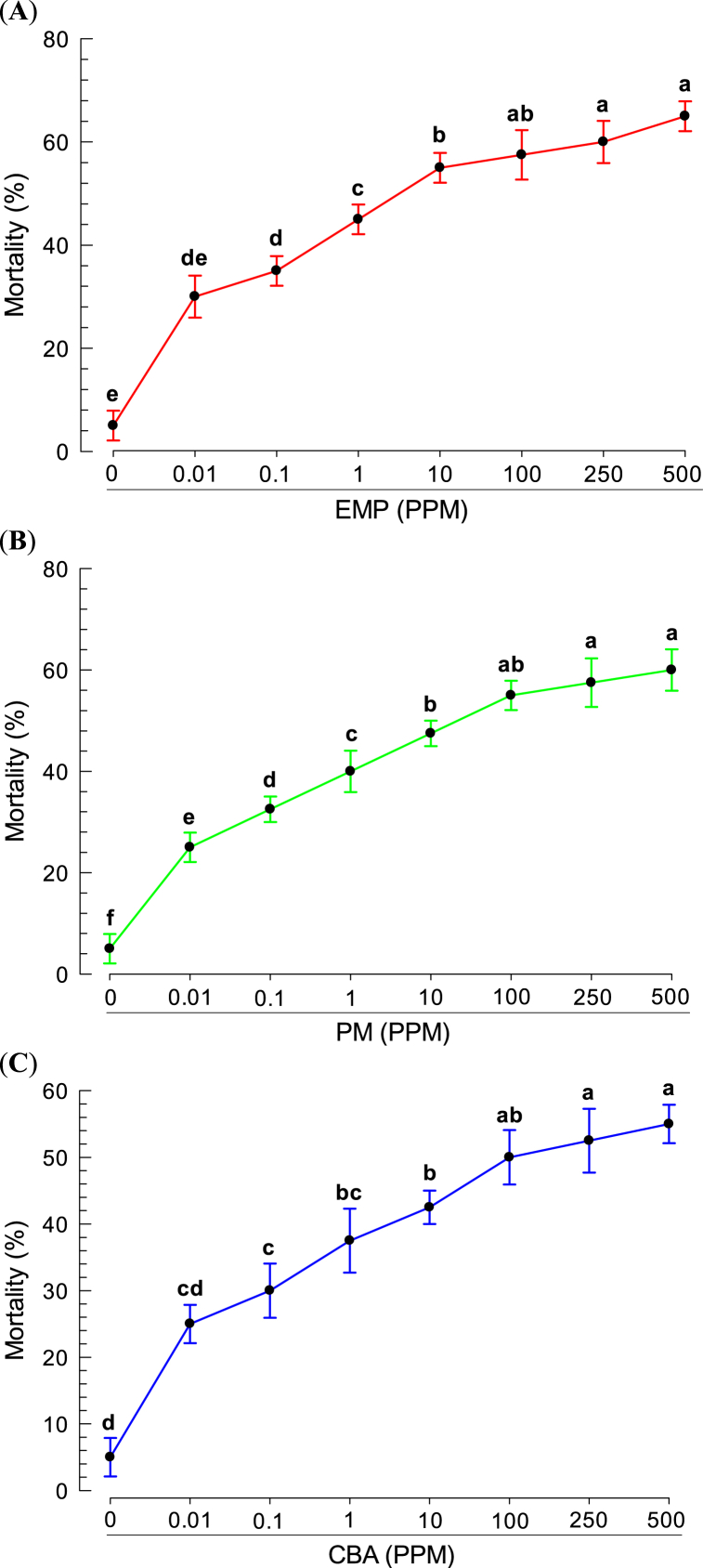
**Insecticidal activity of the predicted DSP1-ligands on *P. xylostella*. (A)** Mortality bio-assay with 3-Ethoxy-4-Methoxyphenol (EMP) **(B)** Mortality bio-assay with Phthalimide **(**PM**)** and **(C)** Mortality bio-assay with O-Cyanobenzoic acid (CBA). *Plutella xylostella* L2 larvae were fed for 24 h on the cabbage leaf that was dipped in the DSP1 ligand solutions of different concentrations. Treated larvae were reared at 25 ± 2 °C temperature and 65 ± 5 % relative humidity. After 24 h, treated cabbage leaves were replaced with fresh leaves. Mortality data was recorded every 24 h up to 96 h. Each treatment was replicated four times and replication consisted of 10 larvae. Different letters above the standard error (SE) indicate significant differences among means at Type I error = 0.05 (LSD test).

Based on the binding affinity of bacterial metabolites to rSe-DSP1, their impact on the survivability of *Plutella xylostella* larvae was analyzed. All three compounds showed significant (*P < 0.0001*) toxicity against the tested larvae. Maximum mortality of *Plutella* larvae was observed in EMP treatment (Fig. 5A). However, PM and CBA also significantly affect the mortality of *Plutella* larvae (Fig. 5B and C). Probit analysis of these compounds revealed that EMP was the most potent with a median lethal dose (LD_50_) of 32.36 ppm while it was 98.04 and 529.25 ppm for PM and CBA, respectively (Table 1).

**Table 1:** Medium lethal dose (LD_50_) of bacterial metabolites.

Compounds	LD_50_ (95% CI)	Slope	SD	SE	χ2	df
EMP	32.36 (0.85–1229.69)	0.190	5.273	0.806	0.990	4
PM	98.04 (3.23–2978.17)	0.204	4.895	0.756	1.00	4
CBA	529.25 (10.16–7265.14)	0.178	5.630	0.876	1.00	4

## 4 Discussion

Damage-associated molecular pattern (DAMP) is the nuclear protein which released in the cytoplasm upon infection or damage [25]. In humans or other vertebrates, HMGB1 plays the DAMP role while DSP1 is the DAMP in insects including *S. exigua* [4,26], *T. molitor* [5], *A. albopictus* [6], and many other insects. The present study identified DSP1 in another lepidopteran insect, *P. xylostella (Px-DSP1)*. Like other DSP1, Px-DSP1 also contains HMG box A, HMG box B, Acidic tail, Coiled coil, and Low complexity. Px-DSP1 has 73.64% sequence similarity with *S. exigua* DSP1 (Se-DSP1) where 97.26 and 97.18% similarity in HMG Box A and HMG Box B, respectively (Fig. S1). These sequence similarities help to hypothesize that the ligands of Se-DSP1 might be the ligand of Px-DSP1. The binding assay of bacterial metabolites to rSe-DSP1 showed that few of the metabolites have a high binding affinity at the very low amount of μM level. A salicylic acid analog, EMP was the most potent in binding to rSe-DSP1. SA is the universal ligand of vertebrate HMGB1 [24]. A previous study by Ref. [9] reported that EMP, PM, and CBA had Kd values of 119, 142, and 166 μM, respectively [18]. reported that metabolites purified from *Xenorhabdus nematophila* and *Photorhabdus temperata temperata* bind to another insect immune protein, sPLA_2_ with a very low Kd value (μM). This study hypothesized that *X. hominickii* metabolites might inhibit the Px-DSP1, a DAMP that induces mortality by host immunosuppression. Another study concluded that EMP treatment in *S. exigua* caused significant immunosuppression [23] and plasma contains no Se-DSP1 [9]. This finding denotes that EMP binds to Se-DSP1 and prevents its translocation from the nucleus. Mortality bioassay with *Plutella* larvae showed that these metabolites can kill more than 65% of insect larvae at 500 ppm concentration. Among the metabolites, EMP with the more binding affinity with DSP1, was more potent to kill the larvae compared to others. Pathogenic bacteria possess a polyketide ketone synthase (PKS) gene cluster responsible for the biosynthesis of virulent factors [27]. Most probably, EMP is a product of PKS [28]. So, Entomopathogens are likely to synthesize and secrete secondary metabolites including EMP, PM, CBA, etc. These metabolites prevent the release of DSP1 from the immune cells which are responsible for immune activation via the Toll/Spz signaling pathway. However, EMP may interact with other immune pathways, receptors, or physiological processes to kill the *P. xylostella* larvae. The present study investigated only DSP1-mediated immunity. Further study is required to optimize the procedure or find out the involvement of any other physiological processes.

## 5 Conclusion

Entomopathogenic bacteria (EPB) kill the insects sharply by secreting different metabolites. Some of these metabolites bind to the immune proteins, dorsal switch protein 1 (DSP1) to nullify the immune activity. DSP1 is conserved in all developmental stages and tissue of *Plutella xylostella*. DSP1 of *Plutella xylostella* and *Spodoptera exigua* have high sequence similarity (73.64%). The binding assay revealed that few bacterial metabolites such as EMP, PM, and CBA have binding affinity to rSe-DSP1 at the micromolar level (148, 197, and 246 μM, respectively). Successive mortality bioassay with these ligands revealed their significant toxicity to *P. xylostella* larvae. These results thus concluded that few bacterial metabolites bind to the insect immune protein DSP1 to inactivate its immune functions. Besides, bacterial metabolites also have toxemia, apoptotic, PLA_2_ inhibitory, or potent insecticidal activity. Upon infection, pathogens apply these weapons to combat against insect's immune activity. However, all these properties can be considered as insect control strategies.

## Funding statement

This research did not receive any specific grant from commercial, public, or non-profit sectors.

## Data availability statement

Data included in article/supplementary material/referenced in article.

## CRediT authorship contribution statement

**Md. Mahi Imam Mollah:** Writing – review & editing, Writing – original draft, Visualization, Validation, Supervision, Software, Resources, Methodology, Investigation, Formal analysis, Data curation, Conceptualization.

## Declaration of competing interest

The authors declare that they have no known competing financial interests or personal relationships that could have appeared to influence the work reported in this paper.
