# Supplementary material for: Ligands of HMG-like dorsal switch protein 1 of Spodoptera exigua leads to mortality in diamondback moth, Plutellaxylostella
Source: Heliyon. 2024 Mar 9;10(6):e27090. doi: 10.1016/j.heliyon.2024.e27090 (PMC10950498; doi:10.1016/j.heliyon.2024.e27090)
Supplement: Multimedia component 1 [file mmc1.docx]

**Figure 5**

**Table S1.** Information ofprimers used in this study.

| **Primer name** | **Sequence** | **Amplicon size** |
| --- | --- | --- |
| Px-DSP1-Forward | GAGGCGCAGGTGAACTACAA | 697 bp |
| Px-DSP1-Reverse | AGTAGAGCCTGCTGTTGAGC |  |

**Table S2.**GenBank accession numbers used for phylogenetic analysis.

| **Acronyms** | **Species** | **Gene** | **Accession number** |
| --- | --- | --- | --- |
| *Mp DSP1* | *Myzuspersicae* | DSP1 | XP_022178944.1 |
| *Rm DSP1* | *Rhopalosiphummaidis* | DSP1 | XP_026806372.1 |
| *Nv DSP1* | *Nasoniavitripennis* | DSP1 | XP_016839160.1 |
| *Hl DSP1* | *Habropodalaboriosa* | DSP1 | KOC71292.1 |
| *Dm DSP1* | *Drosophila melanogaster* | DSP1 | AAN09395.1 |
| *Ad DSP1* | *Anopheles darling* | DSP1 | ETN66707.1 |
| *Cf DSP1* | *Ctenocephalides felis* | DSP1 | XP_026466903.1 |
| *Px DSP1* | *Plutella xylostella* | DSP1 | XP_011560328.1 |
| *Gm DSP1* | *Galleria mellonella* | DSP1 | XP_026748165.1 |
| *Bm DSP1* | *Bombyx mori* | DSP1 | XP_012547112.1 |
| *Se DSP1* | *Spodoptera exigua* | DSP1 | MK 737894 |
| *Ha DSP1* | *Helicoverpa armigera* | DSP1 | XP_021183962.1 |
| *Tc DSP1* | *Tribolium castaneum* | DSP1 | XP_973934.2 |
| *Ld DSP1* | *Leptinotarsa decemlineata* | DSP1 | XP_023011517.1 |
| *Nc DSP1* | *Nephila clavipes* | DSP1 | PRD29151.1 |
| *Tu DSP1* | *Tetranychusurticae* | DSP1 | XP_015795363.1 |
| *Pv DSP1* | *Penaeus vannamei* | DSP1 | XP_027210949.1 |
| *Bp DSP1* | *Brachionusplicatilis* | DSP1 | RNA19426.1 |
| *Eg DSP1* | *Echinococcus granulosus* | DSP1 | XP_024348839.1 |
| *Sr DSP1* | *Strongyloidesratti* | DSP1 | XP_024506421.1 |

LC

Px MGDQGATGGAWGARDEASWWPGGAGELQNQQQLNEEIARSTAASTQQLYTYKMTGGQFTN60

Se MGDRGATGDAWGARDDASWWPGGAGELQHQQQLHEEIARSTAASTHQLYTYKMTGG-FSN59

Gm MGDRGATGGAWGARDESSWWPGGAGELQHQQQLNEEVARSTAAATHQLYTYKMTGG-FSN59

***:****.******::***********:****:**:******:*:********** *:*

LC LC

PxNSSENTSSNYGYRLVS--NNREESPQQQWWYSSGAIDSQQQNSSPTPQNQSSPDAEHGNQ118

Se NGGDNSTPSYDYRIMSNSNAREDSPQQSWWYASGSVESQ-QTSSPTPQGQSSPDSEHNNQ118

Gm NGGDTSTTSYDYRLMPGSNTREESAQQPWWYTSGTVESQ-QTSSPTPQNQSSPDPDQGNQ118

*..:.:: .*.**:: * **:* ** ***:**:::** *.******.***** ::.**

CC

Px QSNGQQNHQQLLQQEQQQNL----------------QQQPL---------------QQAL147

Se QANGIQQNHQTLQQVQQ--EQNQAIQQQQNQLQQQLQQQNLQQAIQQHNRTLQQTLQQQQ176

Gm QANGIQQNHQTLQQQQSQQEQNQAIQQQNQLQQQLQQQQNLQQALQQQSQTLQQSLQQQQ178

*:** *:::* *** *. *** * **

Px QQQNQQQTLQQMLQQHQQQQQS-----------------QQQSQQQQLQQSLQQTLQVSQ190

Se --QSQQETLQQMLQQHQQQQQQQQQHQQ--QQQQQQQQQQQQQQQQALQQSLQQTLQVSQ 232

Gm QQQGQQQTLQQMLQQHQLQQQQQQQQQQQQQQQQQQQQQQQQQQQQALQQSLQQTLQVSQ238

*.**:********** ***. ***.*** *************

CC

Px AQAQVLVQAQQALQQQVAQSLQQQQLSLHDHIQAVQQHQIQAALQRQSATLQELQQQAQQ250

Se AQAQAIVQAQAALQQQVAQTLQQQQQTLHEQLQAVQQQQIQAALQRQSATLQELQHQAQQ292

Gm AQAQALVQAQAALQQQVAQTLQQQQQNLHERMQAVQQQQIQAALQRQSATFQELQQQQ-- 296

****.:**** ********:***** .**:::*****:************:****:*

HMG Box A

PxQALLAQA-TATKGRMPRARAGNKPRGRMTAYAFFVQTCREEHKKKHPDENVIFAAFSKKC309

Se QALLAQAQATVKQKMPRARAYNKPRGRMTAYAFFVQTCREEHKKKHPDENVVFAAFSKKC352

Gm -ALIA-QAAGNKGRMPRARPYNKPRGRMTAYAFFVQTCREEHKKKHPDENVIFAAFSKKC354

**:* : * :***** ******************************:********

PxAERWNTMSEKEKQRFHEMAEHDKKRYDLEMQTYVPPKDVKMGRGRKRHQIKDPNAPKRSL369

Se AERWNTMSEKEKQRFHEMAEQDKRRFDLEMQNYVPPKDMKV-RGRKRQQMKDPNAPKRSL411

Gm AERWNTMSEKEKQRFHEMAEQDKRRYDLEMQNYVPPKDVKV-RGRKR-QVKDPNAPKRSL412

********************:**:*:*****.******:*: ***** *:**********

HMG Box B

PxSAFFWFCNDERSKVKANNPEYTMGDIAKELGRRWAAALPETKTKYEALSEQDKARYDREM429

Se SAFFWFCNDERSKVKANNPEYTMGDIAKELGRRWAAADPETKSKYESLSEQDKARYDREM471

Gm SAFFWFCNDERSKVKASNPMFSMGDIAKELGRLWAAADPETKSKYEALSEQDKARYDREM472

****************.** ::********** **** ****:***:*************

LCAT

PxTAYKKGPLLAAQQAQQQQAAELEEDVGDFEAEDEYN-----465

Se TAYKKGPLALTQQQAAV--VPDVDEDGDFDAEEEYK-----505

Gm TAYKKGHLLAQQQQAQE--VE--DEEGEYLGEGDYETETTA509

****** * ** . :: *:: .* :*:

**Figure S1**
